# Phenotype Overlap and Lung Function in Childhood Asthma: The Interaction Between T2 and Non‐T2 Responses

**DOI:** 10.1002/ppul.71361

**Published:** 2025-10-29

**Authors:** Lívea Gianfrancesco, Ana Paula Gaban Malheiro, Taís Nitsch Mazzola, Icléia Siqueira Barreto, André Moreno Morcillo, Milena Baptistella Grotta, José Dirceu Ribeiro, Adyléia Aparecida Dalbo Contrera Toro

**Affiliations:** ^1^ Child and Adolescent Health Postgraduate Program State University of Campinas Campinas São Paulo Brazil; ^2^ School of Medical Sciences, Center for Investigation in Pediatrics State University of Campinas São Paulo Brazil; ^3^ Department of Pathology, School of Medical Sciences State University of Campinas São Paulo Brazil; ^4^ Department of Pediatrics, School of Medical Sciences State University of Campinas Campinas São Paulo Brazil

**Keywords:** asthma, children, cytokines, phenotype, spirometry, sputum

## Abstract

**Purpose:**

This study aimed to investigate the association between asthma severity, lung function and serum levels of cytokines associated with T‐helper type 2 (T2) and non‐T2 responses in children and adolescents.

**Methods:**

We conducted a cross‐sectional study and recruited children and adolescents (7–17 years old) with asthma. Asthma severity was classified by daily corticosteroid dosage and pulmonary function, while asthma control was assessed using the Asthma Control Test and the Child Asthma Control Test. Spirometry, blood collection for cytokine analysis (IL‐4, IL‐5, IL‐10, IL‐13, IL‐17, IFNγ, TNF‐α), and induced sputum collection for cellularity evaluation were performed.

**Results:**

A total of 80 participants were included, with a mean age of 11.9 years, and 60% were male. Severe asthma was observed in 17.5%, and 15% had uncontrolled asthma. No differences in sputum cellularity were found (*p* = 0.509). Cytokine levels of TNF‐α (*p* = 0.043) and IL‐5 (*p* = 0.010) differed by asthma severity. Negative correlations were observed between cytokines (IL‐4, IL‐5, IL‐10, IL‐17) and spirometric parameters. IL‐17 (*p* = 0.001) and IL‐5 (*p* = 0.045) levels varied with airway obstruction, and IL‐17 levels differed with bronchodilator response in FEV1 (*p* = 0.041).

**Conclusions:**

Our findings highlight the heterogeneity of immune responses in childhood asthma. Cytokines from both T2 and non‐T2 pathways were associated with lung function impairment, underscoring the interplay of diverse immune responses in the complexity of asthma pathophysiology.

## 1 Introduction

Asthma is a complex chronic obstructive disease and a serious global health issue affecting all age groups, with rising prevalence, particularly among children [1]. It significantly impacts children worldwide, with higher mortality and hospitalization rates in low‐ and middle‐income regions [1]. In severe cases, asthma poses increased risks of complications, significant economic burdens, and challenges in treatment management [1].

There is considerable variability in the phenotypes and endotypes of asthma, resulting from mechanisms involving a wide array of inflammatory cells and mediators [2]. It is typically characterized by T‐helper (Th) type 2 (Th2) inflammation mediated by cytokines such as interleukin (IL) 4, IL‐5, and IL‐13, leading to eosinophilic airway inflammation [2]. However, recent studies have indicated that Th17 cells, which primarily induce neutrophilic inflammation, are also closely associated with the inflammatory process in asthma [3, 4].

Smooth muscle contraction and chronic airway inflammation, along with plasma extravasation, edema, and infiltration of inflammatory cells, are primary contributors to airway narrowing. This narrowing results in reduced airflow and clinical symptoms such as wheezing, shortness of breath, cough, and chest tightness [5, 6]. Thus, the assessment of symptoms and pulmonary function are fundamental clinical markers for diagnosing asthma and optimizing its management, ensuring that therapeutic objectives are met [1, 6].

Cytokines play a crucial role in triggering, sustaining, and amplifying the bronchial inflammatory process [5]. Some patients with asthma may experience altered pulmonary function, characterized by reduced or absent airflow variability. Over time, this can result in the loss of bronchodilator responsiveness and the development of fixed airflow obstruction [6, 7].

The inflammatory response and structural changes in the airways associated with asthma are influenced by a range of factors, leading to diverse and evolving findings in the literature [8, 9, 10, 11]. Therefore, utilizing clinical parameters that reflect airway inflammation is crucial for effective asthma management. This study aimed to explore the association between asthma severity, pulmonary function and cytokines related to both T2 (interleukins 4, 5, and 13) and non‐T2 (interleukins 10, 17, tumor necrosis factor‐alpha, and interferon‐gamma) immune responses children and adolescents.

## 2 Methods

A cross‐sectional observational study was conducted and approved by the Ethics Committee of the University of Campinas, under ruling number 6.336.265. Informed consent was obtained from the legal guardians of all participants, and adolescents over 14 years old provided additional assent.

The study included children and adolescents of both sexes, aged 7 to 17 years, with a diagnosis of asthma based on the Global Initiative for Asthma (GINA) [1] guidelines. Recruitment occurred during routine consultations at the Ambulatory Clinics of the University of Campinas Hospital between July 2017 and December 2018. All participants had been under medical care for at least 3 months before the study.

Exclusion criteria included parental request, presence of cardiac or pulmonary comorbidities, and inability to perform any required tests.

Clinical, anthropometric, and demographic data were collected during interviews and medical consultations and were subsequently verified against medical records. Participants were evaluated in accordance with the treatment strategies outlined by GINA [1].

To classify asthma severity, participants were divided into two groups. The criteria used for classification and group division were designed by the authors of this paper. Those classified as having severe asthma met the following criteria: 1) High daily doses of inhaled corticosteroids [1]; 2) presence of airway obstruction, defined as a forced expiratory volume in 1 s to forced vital capacity ratio (FEV_1_/FVC) of < 80% in patients aged 12 years or older and < 90% in patients under 11 years [1]; and 3) an FEV_1_ value of < 80%, based on spirometry data. Participants who did not meet all three criteria were classified as having non‐severe asthma [12].

To evaluate asthma control, the Child Asthma Control Test (c‐ACT) and the Asthma Control Test (ACT) were utilized. These questionnaires assess activity limitations, dyspnea, and nocturnal symptoms experienced over the past 4 weeks, based on the participant's self‐report. Asthma was classified as controlled when the score exceeded 19 and as uncontrolled when the score was ≤ 19 in both questionnaires [13, 14].

In instances of respiratory infections or symptoms of asthma exacerbation occurring up to 15 days before or on the day of the assessment, patients were evaluated by a physician, and the study procedures were rescheduled. All participants were instructed to discontinue their medication the night before the examinations to comply with spirometry guidelines, following recommendations from the European Respiratory Society (ERS) and the American Thoracic Society (ATS) [15].

Spirometry (CPFS/D model, Medical Graphics Corporation, St. Paul, MN, USA) was utilized to assess pulmonary function. All maneuvers were conducted in accordance with the standards set by the American Thoracic Society (ATS) and the European Respiratory Society (ERS) [15]. The variables analyzed included Forced Vital Capacity (FVC), Forced Expiratory Volume in 1 s (FEV_1_), FEV_1_/FVC ratio, Forced Expiratory Flow between 25% and 75% of FVC (FEF_25‐75%_) and Maximum Forced Expiratory Flow (FEFmax). Bronchodilator (BD) response was defined as an increase in FEV_1_ of > 12% and > 200 mL from baseline in adolescents aged 12 years or older, and an increase of > 12% in children aged 7–11 years [1, 16]. In terms of FEF_25%–75%,_ a response was considered as an increase of > 30% from the baseline value post‐bronchodilator [17].

The cutoff point adopted for elevated total IgE was ≥ 96.25 IU/mL, according to Wang et al. [18].

Five milliliters (mL) of peripheral blood were collected from all participants for cytokine measurement. Following processing and centrifugation, the plasma was stored in 80‐microliter aliquots and frozen at −80°C. Serum levels of seven cytokines (IL‐4, IL‐5, IL‐10, IL‐13, IL‐17, IFNγ, TNF‐α) were quantified using the Human High Sensitivity T Cell Magnetic Bead Panel protocol from the MILLIPLEX MAP Kit, according to the manufacturer's instructions. The minimum detectable levels were: IL‐4 = 1.12 pg/mL; IL‐5 = 0.12 pg/mL; IL‐10 = 0.56 pg/mL; IL‐13 = 0.23 pg/mL; IL‐17A = 0.33 pg/mL; IFN‐γ = 0.48 pg/mL; TNF‐α = 0.16 pg/mL. Measurements below the minimum detectable levels were assigned half that value.

The induced sputum samples were collected and analyzed based on the recommendations of the European Respiratory Society (ERS) and were considered satisfactory if they contained ≤ 20% squamous cells, ≥ 50% viable cells, and ≥ 400 inflammatory cells [19]. The profile of inflammatory cells in the sputum was classified as follows: **1)** eosinophilic if eosinophils > 2.5% and **2)** non‐eosinophilic, which included the neutrophilic profile characterized by neutrophils > 54%, mixed granulocytic when eosinophils > 2.5% and neutrophils > 54%, or paucigranulocytic when eosinophils ≤ 2.5% and neutrophils ≤ 54% [20].

### 2.1 Statistical Analysis

All data were collected using standardized paper forms and subsequently entered into the Statistical Package for Social Sciences for Windows, version 22.0 (SPSS Inc., Chicago, IL, USA) and Stata (StataCorp. College Station, USA).

Categorical variables are presented as absolute (N) and relative (%) frequencies. Quantitative variables are described as medians, minimum, and maximum values. Associations between categorical variables were assessed using the chi‐square test or Fisher's exact test, as appropriate. Comparisons of quantitative variables between two independent groups were performed using the Mann–Whitney test. Correlations between quantitative variables were evaluated using Spearman's correlation coefficient.

Cytokine concentrations (IL‐4, IL‐5, IL‐10, IL‐13, IL‐17A, IFN‐γ, and TNF‐α) were log₁₀‐transformed to reduce skewness. Group comparisons (severe vs. non‐severe asthma, presence vs. absence of airway obstruction, and bronchodilator response) were assessed using Mann–Whitney tests, with results displayed in log‐scale boxplots.

A significance level of 5% was adopted for all analyses.

## 3 Results

**Figure 1:**
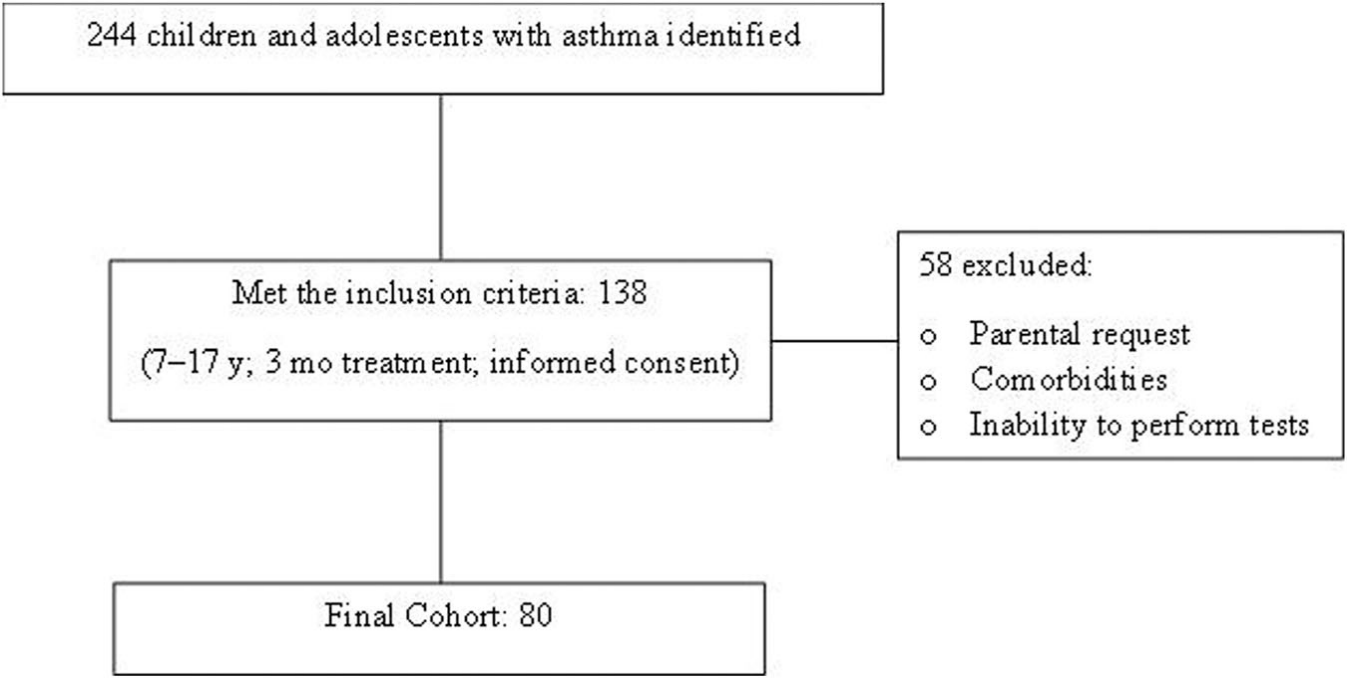
Flowchart of the selection of children and adolescents in the study.

A total of 244 children and adolescents with a diagnosis of asthma were screened for the study, of which 138 were included. Among the included participants, 58 were excluded due to comorbidities, inability to perform the tests, or withdrawal at the time of blood collection, resulting in a final cohort of 80 individuals (Figure 1).

The mean age of the participants was 11.9 ± 2.6 years, with 48 (60%) being male. Among them, 14 (17.5%) had severe asthma and 66 (82.5%) had non‐severe asthma. Twelve participants (15.0%) had uncontrolled asthma. Sputum samples from 54 patients were classified as satisfactory; among the 33 non‐eosinophilic profiles, 29 were paucigranulocytic, 1 mixed, and 3 neutrophilic. Comparison of pre‐bronchodilator pulmonary function between severe and non‐severe asthma patients was not conducted, as this criterion was used for classification of the groups. However, differences were observed between the groups in the FEV_1_ variable during the bronchodilator response evaluation (Table 1).

**Table 1:** Characterization of participants with severe and non‐severe asthma.

	Severe asthma (*n* = 14)	Non‐severe asthma (*n* = 66)	*p*
**Age**	10.42 (8.74–14.65)	12.20 (7.52–17.09)	0.199^a^
**Sex** *n* (%)			
Female	7 (50.0%)	25 (37.9%)	0.400^b^
Male	7 (50.0%)	41 (62.1%)	
**Nutritional status** *n* (%)			
Nonobese	10 (71.4%)	55 (83.3%)	0.286^b^
Obese	4 (28.6%)	11 (16.7%)	
**Asthma control** *n* (%)			
Controlled	12 (85.7%)	56 (84.8%)	0.934^b^
Noncontrolled	2 (14.3%)	10 (15.2%)	
**Sputum cellularity** *n* (%)			
Eosinophilic	4 (40.0%)	17 (38.6%)	0.936^b^
Non‐eosinophilic	6 (60.0%)	27 (61.4%)	
**Total serum IgE**			
≥ 96.25 IU/mL	14 (100.0%)	59 (89.4%)	0.202^b^
< 96.25 IU/mL	0 (0.0%)	7 (10.6%)	
**Pre‐ BD Spirometry**			
FEV_1_ (% predicted)	71 (54–79)	88 (68–117)	
FEV_1_/FVC	73.5 (55.0–86.0)	85.0 (61.0–99.0)	
FEF_25%–75%_ (% predicted)	46 (27–67)	74 (35–141)	
FVC (% predicted)	84 (76–114)	99 (75–132)	
**BD response**			
FEV_1_ BD responders	7 (50.0%)	14 (21.2%)	**0.042** c
FEV_1_ BD nonresponders	7 (50.0%)	52 (78.8%)	

*Note:* Data expressed as absolute and relative frequencies and median (minimum‐maximum).

Abbreviations: D, Bronchodilator; FEV_1_, Forced Expiratory Volume in one second; FVC, Forced Vital Capacity; FEF_25%–75%_, Forced Expiratory Flow between 25% and 75% of FVC; IgE, immunoglobulin E.

^a^ Mann–Whitney test.

^b^ Chi‐square test.

^c^ Fisher's exact test.

**Figure 2:**
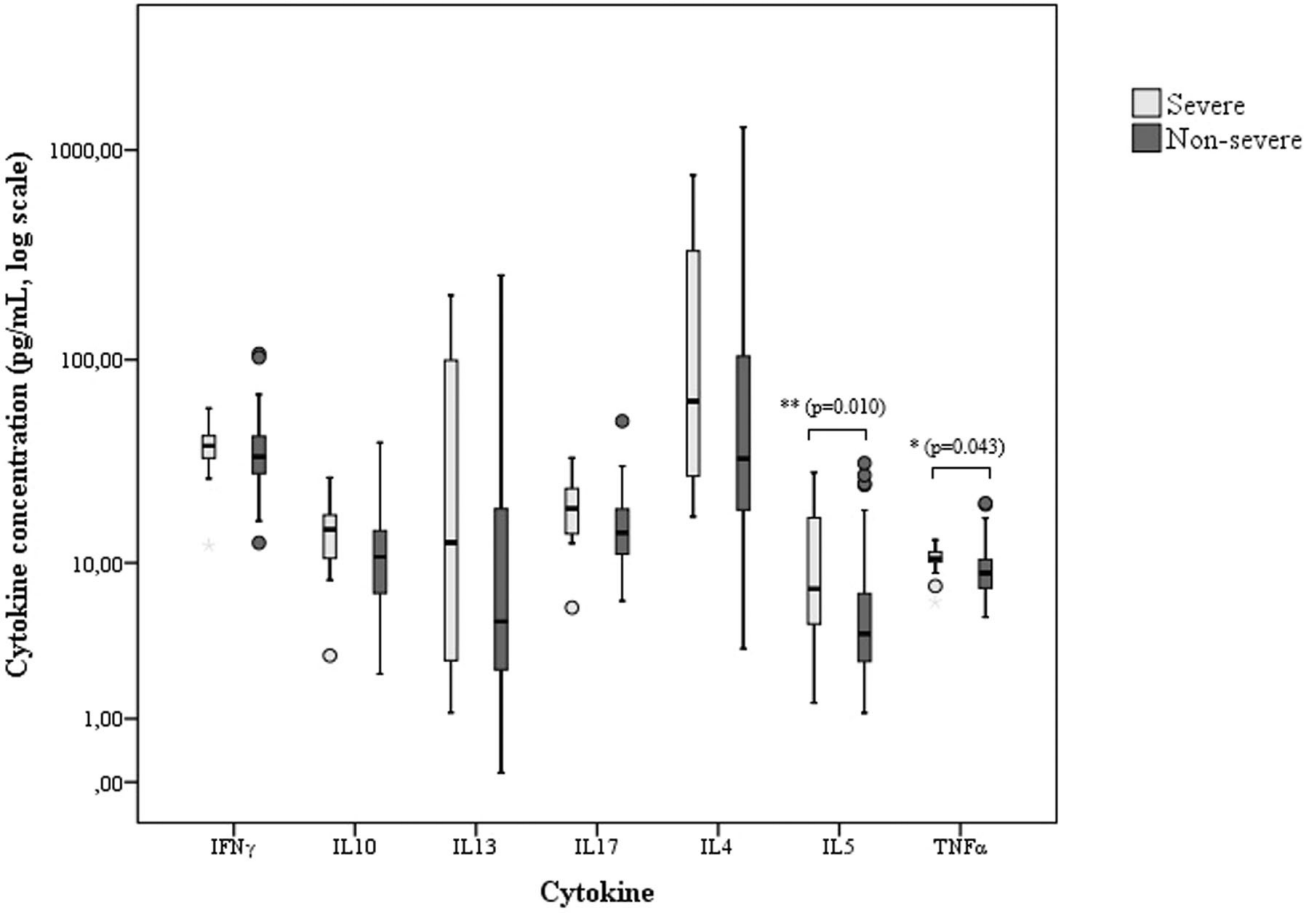
Boxplots of serum cytokine concentrations (pg/mL, log₁₀ scale) according to asthma severity. Boxes represent the interquartile range, horizontal lines indicate the median, and whiskers show the range. Outliers are displayed as circles. Significant between‐group differences were determined using the Mann–Whitney test and are indicated by asterisks: IL‐5 **(*p* = 0.010) and TNF‐α *(*p* = 0.043).

Children with severe asthma had higher median serum levels of TNF‐α (10.56 [6.17–13.14] vs. 8.83 [5.07–20.11] pg/mL, *p* = 0.043) and IL‐5 (7.29 [1.38–28.54] vs. 4.06 [1.13–31.63] pg/mL, *p* = 0.010) compared with those with non‐severe asthma, whereas IFN‐γ, IL‐10, IL‐17, and IL‐4 did not differ between groups (Figure 2). When comparing cytokine concentrations according to asthma control status, no differences were observed between children with controlled and uncontrolled asthma. Among the cytokines measured, only IL‐13 exhibited a minimum observed value below its minimum detectable level.

Upon analyzing the correlation between cytokines and pulmonary function, IL‐10 correlated negatively with FEV_1_ and FVC, IL‐17 with FEV_1_, FEV_1_/FVC, FEF_25%–75%_, and FEFmax. IL‐4 also showed a negative correlation with FEV_1_, FVC, and FEFmax and IL‐5 with FEV_1_, FVC, FEF_25%–75%_, and FEFmax. No correlations were found for IFN‐γ, TNF‐α, or IL‐13 (Table 2).

**Table 2:** Correlation between cytokines (pg/mL) and pre‐bronchodilator pulmonary function tests.

		FEV_1_	FEV_1_/FVC	FVC	FEF_25%–75%_	FEFmax
**IFN**γ	CC	−0.158	−0.057	−0.063	−0.098	−0.163
	*p*	0.163	0.612	0.579	0.387	0.157
**IL10**	CC	−0.251	−0.033	−0.265	−0.109	−0.185
	*p*	**0.025**	0.768	**0.018**	0.336	0.107
**TNF‐α**	CC	−0.100	−0.024	−0.023	−0.093	−0.166
	*p*	0.380	0.834	0.842	0.411	0.149
**IL13**	CC	‐0.166	−0.109	−0.114	−0.152	−0.131
	*p*	0.142	0.336	0.315	0.178	0.258
**IL17**	CC	−0.260	−0.266	−0.021	−0.294	−0.235
	*p*	**0.020**	**0.017**	0.857	**0.008**	**0.040**
**IL4**	CC	−0.238	−0.089	−0.231	−0.154	−0.259
	*p*	**0.034**	0.431	**0.040**	0.174	**0.023**
**IL5**	CC	−0.351	−0.210	−0.232	−0.291	−0.83
	*p*	**0.001**	0.061	**0.038**	**0.009**	**0.013**

*Note:* Spearman's correlation coefficient.

Abbreviations: CC, Correlation coefficient, FEF_25%–75%_, Forced Expiratory Flow between 25% and 75% of FVC; FEFmax, Maximum Forced Expiratory Flow; FEV_1_, Forced Expiratory Volume in one second; FVC, Forced Vital Capacity; IFNγ, Interferon‐gamma; IL, Interleukin; *p*, *p*‐value; TNF‐α, Tumor Necrosis Factor‐alpha.

In secondary analyses, no differences were observed between sputum cellularity and lung function changes (Table 3). However, cytokine distribution varied according to pulmonary function: IL‐17 (*p* = 0.001) and IL‐5 (*p* = 0.045) were associated with the presence of airway obstruction, and IL‐17 also differed according to bronchodilator response in FEV₁ (*p* = 0.041) (Table 3).

**Table 3:** Sputum cellularity and distribution of cytokines (pg/mL) with respect to the presence of airway obstruction and bronchodilator response in the FEV_1_ variable in spirometry.

	Presente of obstruction			FEV_1_ response		
	Yes	No	*p*	Yes	No	*p*
**Sputum cellularity *n*(%)**						
Eosinophilic	15 (69.2%)	7 (31.8%)	0.377^a^	7 (31.8%)	15 (69.2%)	0.270^a^
Non‐eosinophilic	18 (56.2%)	14 (43.8%)		6 (18.8%)	26 (81.2%)	
**IFN**γ	38.27 (12.32–102.65)	31.77 (16.40–106.89)	0.084^b^	39.67 (27.00 – 52.99)	33.45 (12.32 – 106.89)	0.058^b^
**IL10**	11.14 (2.99–38.86)	11.91 (2.27–39.77)	0.558^b^	12.89 (4.43–26.97)	10.73 (2.27–39.77)	0.131^b^
**TNF‐α**	8.88 (5.92–20.11)	9.11 (5.07–19.82)	0.713^b^	8.87 (6.17–13.14)	9.11 (5.07–20.11)	0.995^b^
**IL13**	4.95 (0.11– 253.68)	4.79 (0.11–178.23)	0.202^b^	4.47 (1.71–253.68)	4.81 (0.11–188.62)	0.357^b^
**IL17**	17.09 (5.74–33.52)	12.12 (6.25–50.58)	**0.001** b	18.72 (9.02–30.50)	13.29 (5.74–50.58)	**0.008** b
**IL4**	38.48 (6.57–1281.09)	28.44 (3.31–1012.28)	0.235^b^	35.71 (14.92–1039.66)	31.79 (3.31–1281.09)	0.420^b^
**IL5**	5.08 (1.38– 31.63)	4.00 (1.13–27.67)	**0.045** b	5.87 (1.90–28.54)	4.43 (1.13–31.63)	0.130^b^

*Note:* Data expressed as absolute and relative frequencies and median (minimum‐maximum).

Abbreviations: IFNγ, Interferon‐gamma; IL, Interleukin; TNF‐α, Tumor Necrosis Factor‐alpha.

^a^ Chi‐square test.

^b^ Mann–Whitney test.

## 4 Discussion

According to current guidelines, asthma is a complex and heterogeneous disease encompassing multiple phenotypes [1]. In our cohort, this heterogeneity was reflected by the presence of both eosinophilic and non‐eosinophilic profiles, as well as overlapping inflammatory pathways. Participants were classified based on both treatment level and pulmonary function, since a decline in pulmonary function is often associated with symptomatic asthma and severe exacerbations requiring systemic corticosteroids [21].

In this study, we applied complementary approaches to capture different aspects of asthma pathophysiology. Asthma severity was considered a clinical expression of disease burden, while spirometric parameters—including airway obstruction and bronchodilator response—were used as direct measures of lung function [1]. Notably, bronchodilator response may also reflect underlying inflammatory changes [6, 11]. Serum cytokines provided insight into systemic inflammation, and induced sputum samples reflected airway cellular inflammation [10, 22]. Together, these analyses aimed to clarify how disease severity and functional impairment relate to T2 and non‐T2 pathways in childhood asthma.

When analyzing cytokine distribution according to asthma severity, we found higher median concentrations of TNF‐α in the severe‐asthma group compared with the non‐severe group (2.8 vs. 1.9 pg/mL; *p* = 0.043). TNF‐α is a pro‐inflammatory cytokine that attracts neutrophils and eosinophils and has been implicated in the pathophysiology of various chronic inflammatory diseases [23]. Berry et al. demonstrated that TNF‐α upregulation is associated with severe, refractory asthma, involving increased neutrophil recruitment and airway inflammation [24], while Aldhalmi et al. suggest that individuals with mild asthma who are managed with low doses of inhaled corticosteroids are likely to have suppressed TNF‐α production [25].

In addition to the difference observed in TNF‐α between severe and non‐severe asthma, median IL‐5 concentrations were also higher in the severe group (1.7 vs. 0.8 pg/mL; *p* = 0.010). IL‐5 is a potent pro‐inflammatory cytokine that regulates the maturation, proliferation, activation, and migration of eosinophils in asthma, which are critically implicated in asthma severity [26, 27]. These findings are consistent with previous evidence demonstrating the role of eosinophils and their mediators in disease burden [26, 27].

By contrast, no differences in cytokine distribution were observed between children with controlled and uncontrolled asthma. Rogers et al., however, reported that children with uncontrolled asthma had higher serum levels of IL‐10, IL‐13, and TNF‐α, differing from our findings [28]. Their study evaluated children with and without obstructive sleep apnea, suggesting that comorbid conditions may contribute to variability in inflammatory patterns [28].

Guidelines recommend adjusting asthma management based on disease control, which is typically assessed through factors such as daytime symptoms, nighttime awakenings, limitations in activity, use of β2‐agonists as rescue medication, and the frequency of emergency visits and hospitalizations [1]. However, symptom perception is influenced by various factors, and not all patients are able to accurately perceive symptoms of airflow limitation [29].

Cytokines play a crucial role in the inflammatory and pathophysiological processes associated with airway obstruction in asthma, with the airway epithelium serving as both mediator and target of inflammation [11]. Supporting this, Puneeth et al. identified FEV₁ as the most appropriate spirometric index for assessing disease severity in children with asthma [30].

When analyzing the correlation between cytokines and pulmonary function, IL‐10 showed negative associations with FEV₁ (*ρ* = –0.251; *p* = 0.025) and FVC (*ρ* = –0.265; *p* = 0.018), suggesting that higher IL‐10 concentrations may be associated with reduced pulmonary function in children and adolescents with asthma. IL‐10 is a potent anti‐inflammatory cytokine that negatively regulates the synthesis of Th1 and Th2 cytokines, chemokines, and inflammatory enzymes [31, 32]. However, John et al. have noted that pro‐inflammatory cytokines and corticosteroid use can lead to a secondary increase in IL‐10 release [32].

Similarly, IL‐17 showed negative correlation with FEV₁ (*ρ* = –0.260; *p* = 0.020), FEV₁/FVC (*ρ* = –0.266; *p* = 0.017), FEF₂₅%–₇₅% (*ρ* = –0.294; *p* = 0.008), and FEFmax (*ρ* = –0.235; *p* = 0.040). IL‐17 is a pro‐inflammatory cytokine that plays a central role in the activation of neutrophils and macrophages in the lungs of patients with severe asthma [3, 4]. According to the systematic review by Hofmann et al., which assessed the role of IL‐17 in atopy, elevated serum levels of IL‐17 were consistently observed in studies involving both adults and children with allergic asthma [33]. Furthermore, a direct association has been established between IL‐17 and chronic airway inflammation, as well as airway remodeling processes in severe asthma [33].

In addition to IL‐10 and IL‐17, interleukins 4 (IL‐4) and 5 (IL‐5) were also negatively correlated with pulmonary function. Both cytokines are crucial in the cascade of events leading to airway inflammation in asthma. IL‐5 is essential for the recruitment and survival of eosinophils, while IL‐4 may contribute significantly to phenotypic or functional changes in asthma, such as airway hyperresponsiveness, eosinophil infiltration, and mucus overproduction, potentially exacerbating airway inflammation [2, 22, 34, 35].

Complementing these findings, IL‐5 was associated with the presence of airway obstruction in our cohort, consistent with observations by Lee et al., who reported that elevated IL‐5 levels are linked to severe airway obstruction [36]. According to Scott et al., although eosinophilic inflammation has been associated with pulmonary function decline, there is no conclusive evidence directly linking IL‐5 to overall impairment in asthma, making its association with obstruction an important finding in our study [11]. Interestingly, we observed a relationship between IL‐17 and both airway obstruction and bronchodilator response in FEV1 values. The reduction in airway patency associated with IL‐17‐mediated inflammation may contribute to the presence of obstruction [37].

According to most studies in the literature, Th17 cells and their cytokines are implicated in neutrophilic inflammation processes. It is suggested that these cells may contribute to the development of certain subgroups of corticosteroid‐resistant asthma [3, 4]. However, our study identified a relationship between IL‐17 and bronchodilator response. The study by Busse et al. emphasized the direct role of IL‐17 in smooth muscle contraction, demonstrating clinically significant effects of anti‐IL‐17A in a cohort with high FEV_1_ reversibility in response to salbutamol. Notably, the authors did not measure IL‐17 levels in their studied population [38].

The simultaneous detection of T2 and non‐T2 cytokines in our cohort not only reflects the broad spectrum of phenotypes but also highlights a critical insight into asthma's heterogeneous nature. Our findings suggest that the rigid classification of asthma phenotypes may not fully capture the complexity of the disease in many patients [39, 40]. Although eosinophilic inflammation appeared predominant—consistent with the absence of a significant neutrophilic pattern in induced sputum—the coexistence of T2 markers (such as IL‐4, IL‐5, and elevated IgE) with non‐T2 cytokines (including IL‐17, TNF‐α, and IFN‐γ) indicates that multiple inflammatory pathways contribute to disease expression.

This study has some limitations. First, the sample size may restrict the generalizability of the findings. Second, the participants’ IgE samples were not collected on the same day as the cytokines and induced sputum. Third, not all cytokines potentially associated with the inflammatory process were assessed.

Our results highlight the complexity of inflammation in childhood asthma. Although only one participant exhibited a mixed cellular profile in induced sputum, elevated IgE and other markers of T2 inflammation frequently coexisted with non‐T2 cytokines (IL‐17, TNF‐α, IFN‐γ). The severity of the disease was notably associated with pro‐inflammatory cytokines such as TNF‐α and IL‐5, adding to the growing evidence that asthma cannot be understood through a single phenotypic lens.

Taken together, our results highlight that both T2 and non‐T2 cytokines play a critical role in modulating airway inflammation and affecting pulmonary function in childhood asthma. Personalized approaches that consider the variability of inflammatory profiles may be necessary for more effective management of asthma in children and adolescents.

## Author Contributions

**Lívea Gianfrancesco:** conceptualization, investigation, writing – original draft, methodology, writing – review and editing, validation, visualization, software, formal analysis, project administration, data curation, supervision, resources. **Ana Paula Gaban Malheiro:** investigation, methodology, visualization, writing – review and editing. **Taís Nitsch Mazzola:** conceptualization, methodology, writing – review and editing, data curation. **Icléia Siqueira Barreto:** methodology, writing – review and editing, data curation. **André Moreno Morcillo:** conceptualization, writing – review and editing, software, formal analysis. **Milena Baptistella Grotta:** conceptualization, writing – review and editing, visualization, project administration. **José Dirceu Ribeiro:** conceptualization, visualization, writing – review and editing. **Adyléia Aparecida Dalbo Contrera Toro:** conceptualization, investigation, writing – original draft, methodology, validation, visualization, writing – review and editing, project administration, supervision, resources.

## Ethics Statement

This study was approved by the Research Ethics Committee of the Faculty of Medical Sciences of the State University of Campinas, ruling no. 6.336.265. The informed consent form was signed by the participants’ legal guardians and adolescents aged over 14 years signed an additional assent form.

## Conflicts of Interest

The authors declare no conflicts of interest.

## Data Availability

The data that support the findings of this study are available on request from the corresponding author. The data are not publicly available due to privacy or ethical restrictions.
